# Osteosarcopenia, an Asymmetrical Overlap of Two Connected Syndromes: Data from the OsteoSys Study

**DOI:** 10.3390/nu13113786

**Published:** 2021-10-26

**Authors:** Maryam Pourhassan, Bjoern Buehring, Ulrik Stervbo, Sven Rahmann, Felix Mölder, Sebastian Rütten, Ulrike Trampisch, Nina Babel, Timm Henning Westhoff, Rainer Wirth

**Affiliations:** 1 Department of Geriatric Medicine, Marien Hospital Herne, Ruhr-University Bochum, Hölkeskampring 40D, 44625 Herne, Germany; ulrike.trampisch@elisabethgruppe.de (U.T.); Rainer.Wirth@elisabethgruppe.de (R.W.); 2 Rheumazentrum Ruhrgebiet, Ruhr-University Bochum, 44649 Herne, Germany; Bjoern.Buehring@elisabethgruppe.de; 3 Center for Translational Medicine and Immune Diagnostics Laboratory, Medical Department I, Marien Hospital Herne, Ruhr-University Bochum, 44625 Herne, Germany; ulrik.stervbo@elisabethgruppe.de (U.S.); nina.babel@elisabethgruppe.de (N.B.); timm.westhoff@elisabethgruppe.de (T.H.W.); 4 Algorithmic Bioinformatics, Center for Bioinformatics, Saarland University, 66041 Saarbrücken, Germany; Sven.Rahmann@uni-due.de; 5 Algorithms for Reproducible Bioinformatics, Genome Informatics, Institute of Human Genetics, University Hospital Essen, University of Duisburg-Essen, 45147 Essen, Germany; felix.moelder@uni-due.de; 6 Institute of Pathology, University Hospital Essen, University of Duisburg-Essen, 45147 Essen, Germany; 7 Center for Orthopedics and Trauma Surgery, St. Anna Hospital, St. Elisabeth Gruppe, 44649 Herne, Germany; sebastian.ruetten@elisabethgruppe.de; 8 Charité—Universitätsmedizin Berlin, Corporate Member of Freie Universität Berlin, Humboldt-Universität zu Berlin, Berlin Institute of Health, Berlin-Brandenburg Center for Regenerative Therapies, 10117 Berlin, Germany

**Keywords:** osteopenia, osteoporosis, sarcopenia, osteosarcopenia, bone mineral density, muscle mass

## Abstract

Osteoporosis and sarcopenia are two chronic conditions, which widely affect older people and share common risk factors. We investigated the prevalence of low bone mineral density (BMD) and sarcopenia, including the overlap of both conditions (osteosarcopenia) in 572 older hospitalized patients (mean age 75.1 ± 10.8 years, 78% women) with known or suspected osteoporosis in this prospective observational multicenter study. Sarcopenia was assessed according to the revised definition of the European Working Group on Sarcopenia in Older People (EWGSOP2). Low BMD was defined according to the World Health Organization (WHO) recommendations as a T-score < −1.0. Osteosarcopenia was diagnosed when both low BMD and sarcopenia were present. Low BMD was prevalent in 76% and the prevalence of sarcopenia was 9%, with 90% of the sarcopenic patients showing the overlap of osteosarcopenia (8% of the entire population). Conversely, only few patients with low BMD demonstrated sarcopenia (11%). Osteosarcopenic patients were older and frailer and had lower BMI, fat, and muscle mass, handgrip strength, and T-score compared to nonosteosarcopenic patients. We conclude that osteosarcopenia is extremely common in sarcopenic subjects. Considering the increased risk of falls in patients with sarcopenia, they should always be evaluated for osteoporosis.

## 1. Introduction

Osteoporosis and sarcopenia are two chronic conditions, which widely affect older people and share a common pathophysiology [1]. Both entities are associated with an increased risk of fractures and other complications and create a significant economic and social burden for the health care system [2,3,4]. Osteoporosis is a systemic bone disease characterized by decreased bone mineral density (BMD) and degraded microarchitecture, resulting in the development of fragility fractures [5]. By contrast, sarcopenia is a muscle disease characterized by loss of muscle mass and muscle strength [6]. Notably, bone and muscle tissue show strong interactions throughout life [7]. Osteoporosis and sarcopenia share common risk factors and contribute to a variety of negative health outcomes, such as falls and fractures, poor mobility, hospital admissions, morbidity, and mortality [8,9,10,11,12,13].

Evaluation of sarcopenia and osteoporosis and their association requires a clear definition for both conditions. Despite consensus regarding the definition of osteoporosis since 1994 [14], there is no universally accepted definition and diagnostic procedure for sarcopenia. Therefore, it is not surprising that prevalence estimates show great variation between studies and even differ by up to 40% with the application of different diagnostic criteria into the same population [15,16,17]. However, the recently updated definition of the European Working Group of Sarcopenia for Older People (EWGSOP2) [18] is increasingly accepted and therefore applied in this study.

There is growing evidence of an interrelationship between low BMD and sarcopenia, and their association has been demonstrated in a meta-analysis [19]. Both conditions may partly overlap and may be present in the same patient. Therefore, the term osteosarcopenia has been proposed to describe this overlap [2,10,19]. Osteosarcopenia is a unique syndrome that exacerbates negative health outcomes, causing a loss of independence and poor quality of life, especially in frail subjects [2,10]. Although there are some prevalence data from geriatric inpatients, community-dwelling older individuals, or patients with a history of hip fractures [20,21,22], the prevalence of osteosarcopenia has not yet been fully researched. Therefore, we sought to investigate the prevalence of low BMD and sarcopenia and the overlap between both conditions in hospitalized patients of the OsteoSys study.

## 2. Methods

The OsteoSys study is a prospective observational multicenter study designed to investigate osteoporosis in the context of chronic inflammation and cardiovascular complications. All consecutively hospitalized patients with known or suspected osteoporosis and willing to participate in this study were recruited from three hospitals in Herne, Germany (Center for Orthopedics and Trauma Surgery, St. Anna Hospital (STA); Rheumatology Center Herne (RZR); and Marien Hospital Herne (MHH)) in the period from February 2017 to October 2019. Written informed consent was obtained from all patients. The study protocol was approved by the ethical committee of Ruhr University Bochum (no. 16-5714 approved 07.06.16, including an amendment approved 24.01.2017). 

### 2.1. Geriatric Assessment

All assessments were made during the first days after hospital admission. Frailty was diagnosed based on the FRAIL scale [23] with a score of 0 being not frail, 1–2 prefrail, and 3–5 frail. The risk of sarcopenia was evaluated based on the SARC-F questionnaire [24] with a total score of 10, and subjects with score ≥4 were defined as having probable sarcopenia. Physical performance was measured with the short physical performance battery (SPPB) [25] with 8 points or lower indicating impaired physical performance. Handgrip strength was assessed with a Jamar-type dynamometer (Lafayette Instrument Company, Lafayette, LA, USA, in MHH and Leonardo Mechanograph GF, Novotec Medical GmbH, Pforzheim, Germany, in RZR and STA). Handgrip strength was measured three times at the dominant or unaffected side, and the maximum score was recorded. All participants were asked whether or not there was a pre-existing diagnosis of osteoporosis. 

### 2.2. Diagnosis of Sarcopenia

Sarcopenia was assessed according to the revised definition of the European Working Group on Sarcopenia in Older People (EWGSOP2) [18] comprising handgrip strength, appendicular muscle mass, and SPPB. Cut-off points of <27 kg for men and <16 kg for women were applied to define low handgrip strength. Appendicular muscle mass (ASM), as measured by dual-energy X-ray absorptiometry (DXA, GE Medical Systems, Madison, WI, USA), was calculated as the sum of lean mass in the arms and legs. The cut-offs used to define low muscle mass were set at ASM <20 kg for men and <15 kg for women. The short physical performance battery (SPPB) was applied for evaluating lower extremity function, and a cut-off score of ≤8 points was considered as low physical performance in both genders. According to the EWGSOP2 consensus, sarcopenia was defined as probable when low muscle strength was detected. A sarcopenia diagnosis was confirmed by the presence of low muscle strength and low ASM. Sarcopenia was considered severe when low handgrip strength, low ASM, and low SPPB were all detected. Patients with normal handgrip strength and normal ASM were classified as nonsarcopenic.

### 2.3. Diagnosis of Low BMD 

A DXA scan was performed with Lunar Prodigy Advance (GE Medical Systems, Madison, WI, USA) in all three centers to measure bone mineral density (BMD) and body composition. Patients were in a supine position, and BMD was measured at three sites in the same session, namely lumbar spine, total femur, and femoral neck. Quality assurance was done on a regular basis, and CV% was less than 1 for all measurement cites. In accordance with the German and other osteoporosis guidelines [26,27], the lowest T-score of the femoral neck, the femur, or the average value of the lumbar vertebrae 1–4 was used for the diagnosis. Vertebral bodies with hardware, degenerative changes, or fractures were excluded according to available guidelines. Every single DXA measurement was validated by an osteoporosis expert (BB). Osteoporosis was defined according to the World Health Organization (WHO) recommendations [28] as BMD of ≤−2.5 standard deviation (SD) below the values of a young healthy adult reference population (T-score) and osteopenia as BMD between −1.0 and −2.5 standard deviations. Low BMD was defined as T-score < −1.0.

### 2.4. Diagnosis of Osteosarcopenia

Osteosarcopenia was diagnosed when both low BMD and sarcopenia were present.

### 2.5. Data Analysis

The statistical analysis was performed using SPSS statistical software (SPSS Statistics for Windows, IBM Corp., Version 27.0, Armonk, NY, USA). Continuous variables are expressed by means and standard deviations (SDs) for normally distributed variables and median values with interquartile ranges (IQR) for non-normally distributed data. Categorical variables are shown as absolute numbers and percentages (n, %). A group comparison was performed using the unpaired *t*-test for continuous data with normal distribution, the Mann–Whitney *U* test for continuous variables with non-normal distribution, and Pearson chi-square test for categorical variables. A Venn diagram illustrates the overlap between sarcopenia and low BMD. In addition, binary logistic regression analyses were used to investigate whether the presence of sarcopenia increases the risk of presenting low BMD and vice versa. Statistical significance was set at *p* < 0.05.

## 3. Results

### 3.1. Characteristics of Study Participants 

A total of 890 patients (age range 59–97 years, 714 females (80%)) participated in the OsteoSys study. Body composition measured by DXA was not available for 318 patients. Patients without whole-body DXA were not included in this analysis because sarcopenia cannot be confirmed without appendicular muscle mass according to the EWGSOP2 definition. Finally, 572 patients (150 patients from RZR, 139 patients from STA, and 283 patients from MHH) with a mean age of 75.1 ± 10.8 years (78% females) had a dataset allowing the diagnosis of sarcopenia and low BMD and were therefore included in this study. SPPB was missing in 270 patients due to acute disease. Baseline characteristics of study participants are summarized in Table 1. Major reasons of hospitalization were cardiovascular disease, poststroke care, pneumonia, urinary tract infection, osteoporosis, falls and fractures, osteoarthritis, and rheumatologic diseases. Using the frail simple score, 41% (n = 226) of participants were classified as frail, while 49% (n = 272) were regarded as prefrail, with the remaining 11% (n = 60) being nonfrail. According to SARC-F, 59% (n = 331) had probable sarcopenia. SPPB was measured in 302 patients in which almost half of the participants (49%, n = 149) showed poor physical performance (SPPB ≤ 8). Further, 20% (n = 111) of the participants reported a previously known osteoporosis.

### 3.2. Prevalence of Sarcopenia and Low BMD

**Figure 1:**
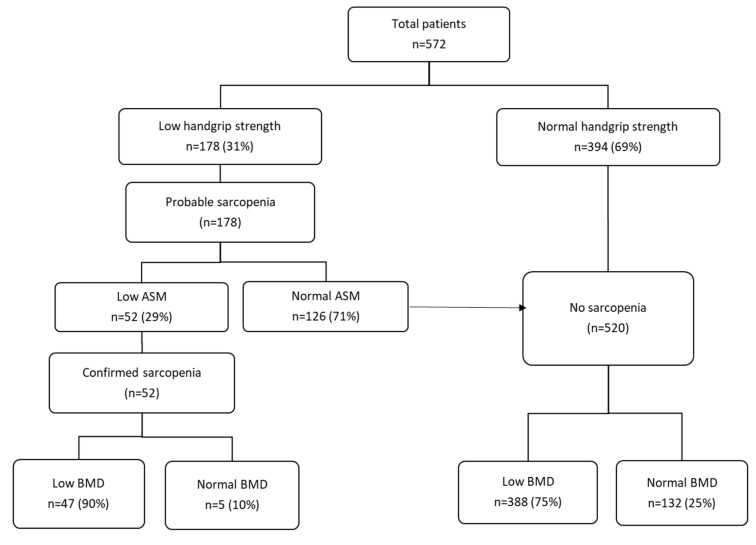
Identifying patients with sarcopenia and low BMD in total population (n = 572). ASM, appendicular skeletal muscle mass; BMD, bone mineral density. Sarcopenia is probable when low muscle strength, as measured by handgrip strength, is detected. A sarcopenia diagnosis is confirmed by the presence of low ASM, as measured by DXA and low muscle strength. Patients with normal handgrip strength and normal ASM were classified as non-sarcopenic.

**Figure 2:**
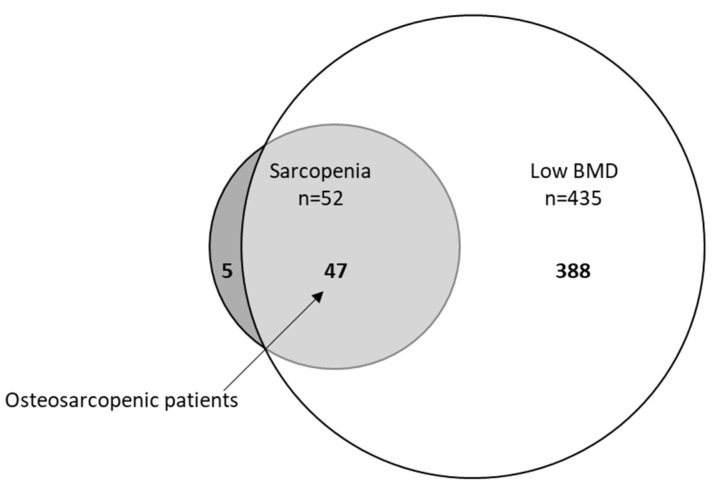
Venn diagram displaying extent of overlap between sarcopenia and low BMD (bone mineral density) in total population (n = 572). Small circle: sarcopenia (52/572, 9%), big circle: low BMD (435/572, 76%), light grey osteosarcopenia patients (47/572, 8%).

Results from the assessment of sarcopenia are given in Figure 1. All included patients were assessed for their lean mass and handgrip strength. Out of 572 patients, 394 patients (69%) had normal handgrip strength and were classified as nonsarcopenic, and 178 patients (31%) had low handgrip strength and were classified as probable sarcopenic according to the criteria of EWGSOP2. By the presence of low ASM, sarcopenia was confirmed in 52 patients (29%, 52/178), of which 25 patients (76%) had low SPPB and fulfilled the criteria for severe sarcopenia (of those with sarcopenia, SPPB values were missing for 19 patients). Out of 178 patients with probable sarcopenia, 126 patients had normal muscle mass and were classified as nonsarcopenic. In addition, 90% (n = 47) of those who were sarcopenic had a low BMD compared to 75% (n = 388) of nonsarcopenic patients (*p* = 0.01). By contrast, only 11% of patients with low BMD demonstrated sarcopenia. The overlap between sarcopenia and low BMD in total population is illustrated in a Venn diagram in Figure 2. In all, 9% (52/572) were sarcopenic, 76% (435/572) had low BMD (osteopenia 245/572, 43%; osteoporosis 190/572, 33%), and 8% (47/572) demonstrated osteosarcopenia (i.e., both low BMD and sarcopenia). 

### 3.3. Comparison of Patients with and without Sarcopenia 

The differences between sarcopenic and nonsarcopenic patients are given in Table 2. Patients with sarcopenia were older and frailer and displayed lower body weight, height, BMI, handgrip strength, SPPB score, body composition components such as MM of the arms and legs, RSMI, FM, and T-score and worse SARC-F score than those without sarcopenia. In a binary logistic regression, low BMD (*p* = 0.048) and age (*p* = 0.002) were significantly associated with sarcopenia. In contrast, age (*p* < 0.002) was the only significant predictor associated with low BMD.

### 3.4. Comparison of Patients with Low and Normal BMD

Compared to the patients with normal BMD, patients with low BMD were older and had lower body weight, height, BMI, handgrip strength, body composition components, and T-score (Table 3).

### 3.5. Comparison of Sarcopenic and Nonsarcopenic Patients with Normal and Low BMD

Compared to sarcopenic patients with normal BMD, sarcopenic patients with low BMD (osteosarcopenia) had significantly lower body weight, MM of the arms, RSMI, and T-score (Table 4). By contrast, nonsarcopenic patients with low BMD were older and displayed lower body weight, height, BMI, handgrip strength, body composition components such as MM of the arms and legs, RSMI, FM, and T-score than those with normal BMD (Table 4).

### 3.6. Comparison of Patients with and without Osteosarcopenia

Osteosarcopenic patients were older and frailer and had lower body weight, height, BMI, handgrip strength, SPPB score, body composition components, and T-score and worse SARC-F score compared to nonosteosarcopenic patients (Table 5).

## 4. Discussion

Despite the fact that low BMD and sarcopenia are highly prevalent in older individuals, both remain underdiagnosed and undertreated [29]. Bone and muscle, which are the main components of the musculoskeletal system, represent 55% of a healthy person’s body mass and show strong interactions [7,29]. With advancing age, a progressive decline in muscle and bone mass is observed. Several studies have shown that low BMD and sarcopenia, which are two major musculoskeletal diseases, are interrelated [19,20,30]. The results of this study demonstrate that low BMD, according to WHO criteria, was prevalent in 76% of the study population with suspected osteoporosis. In addition, the prevalence of sarcopenia was 9% of the total population, with 90% of the sarcopenic patients showing the overlap of osteosarcopenia (8% of the entire population).

The prevalence rate of sarcopenia is generally dependent on the definition used as well as the population studied and is estimated to affect between 10 and 50% of older hospitalized patients [12,31,32,33] and between 1 and 29% of community-dwelling older adults [34]. The prevalence found in our study was different compared to the published works. In a cross-sectional study by Reiss et al. [35], with a sample of 144 geriatric inpatients (mean age 80.6 ± 5.5 years), the EWGSOP2 criteria yielded a prevalence rate of 18%, which is two times higher than our findings. Moreover, using the criteria of EWGSOP2, some estimates are as high as 23% among older hospitalized patients [36,37]. Despite using the same general definition for assessing sarcopenia in these studies, different prevalence estimates were seen. The discrepancy might be due to the operationalization of sarcopenia, different techniques to measure muscle mass, different criteria used to define reduced muscle mass and function, and different study populations [16,38]. For example, in the study by Reiss et al. [35], the participants were older and more often cognitively impaired than in our case. In addition, bioelectrical impedance analysis (BIA) was used for quantification of muscle mass, which is less precise, and timed up and go test (TUG) was performed for evaluating physical performance, both different from our study.

The major finding of this study is that nearly every patient with sarcopenia also suffered from low BMD (90%). Accordingly, we found a statistically significant lower mean BMD in sarcopenic compared to nonsarcopenic individuals, which is in agreement with the results of a systematic review and meta-analysis [19]. Conversely, only few patients with low BMD demonstrated sarcopenia (11%), representing a remarkable asymmetrical overlap. Our findings are in line with a cohort study among 1099 older men and women aged ≥60 years, which demonstrated that the majority of patients with sarcopenia had osteoporosis (57.8%) but only few patients with osteoporosis had sarcopenia (19.1%) [39]. Similarly, in a cross-sectional study among 288 older subjects aged 74.7 ± 5.7 years, 81.1% of sarcopenic patients had osteopenia, whereas only 18.1% of osteopenic subjects had sarcopenia [40]. The asymmetrical overlap seen in our study and previous works suggests that patients with sarcopenia should be routinely evaluated for the probable co-occurrence of osteoporosis. Considering the increased risk of falls and fractures in sarcopenic subjects [41,42], this could be a reasonable strategy to decrease the risk of fractures in this high-risk population.

Further, lower muscle mass and muscle strength (i.e., two of the three components in sarcopenia diagnosis) were observed in patients with low BMD compared to normal BMD, reflecting the shared pathophysiology of both syndromes, such as malnutrition and low physical activity. Accordingly, we found significant associations between osteosarcopenia and lower handgrip strength, worse SPPB, lower muscle mass, and frailty compared to nonosteosarcopenic subjects. Findings of a cross-sectional study among 253 community-dwelling older adults (mean aged 77.9 years) revealed that subjects with osteosarcopenia had greater impairment of physical performance, balance, and limit of stability compared to the nonosteosarcopenic group [43]. In a population of 68 prefrail individuals, Drey et al. [8] reported that osteosarcopenic patients had lower handgrip strength than nonosteosarcopenic patients, which is again in line with our results. All together, these data suggest that osteosarcopenia is a common condition, particularly in older adults, that is associated with more functional impairment than low BMD or sarcopenia alone. In this context, osteosarcopenia seems to be the advanced stage of both syndromes.

Some limitations of the present study should be mentioned. This was a cross-sectional study design, so it did not allow the association with outcome measures to be analyzed. Almost one-third of the study population was not included in the analysis due to missing DXA measurements. In addition, physical performance, as measured by SPPB, was missing in half of the study population, most likely due to immobility related to acute disease. Further, our sample with known or suspected osteoporosis, which is composed of geriatric, orthopedic, and rheumatologic patients, was not representative for general hospitalized patients. 

## 5. Conclusions

Osteosarcopenia, the overlap of low BMD and sarcopenia, is extremely common in sarcopenic subjects. Considering the increased risk of falls in patients with sarcopenia, they should always be evaluated for osteoporosis.

## Figures and Tables

**Table 1:** Characteristic of study population.

	Total Population (n = 572)
Gender (n, %)	
Female	449 (78)
Male	123 (22)
Age (year)	75.1 ± 10.8
Height (m)	1.64 ± 0.08
Actual body weight (kg)	74.0 ± 15.6
BMI (kg/m^2^)	27.3 ± 5.3
Geriatric assessment	
Handgrip strength (kg)	22.6 ± 10.8
Frail scale, median (IQR)	2 (1–3)
SARC-F scores, median (IQR)	4 (2–6)
SPPB, median (IQR)	9 (5–10)
Body composition	
MM_arms_ (kg)	4.5 ± 1.2
MM_legs_ (kg)	13.9 ± 3.0
AMM (kg)	18.4 ± 4.1
RSMI (kg/m^2^)	6.8 ± 1.2
Body fat (%)	39.8 ± 8.1
Bone mass density	
T-score	−1.8 ± 1.3

SPPB, short physical performance battery; MM_arms_, muscle mass of the arms; MM_legs_, muscle mass of the legs; AMM, appendicular muscle mass (muscle mass of the arms + muscle mass of the legs); RSMI, relative skeletal muscle mass index. Frail simple, SARC-F, and SPPB were measured in 558, 561, and 302 subjects, respectively. Values are given as mean ± SD, median (interquartile range), or number (%).

**Table 2:** Comparison of sarcopenic and nonsarcopenic patients.

	Sarcopenia n = 52 (9%)	No Sarcopenia n = 520 (91%)	*p* Value
Gender (n, %)			
Female	38 (73)	411 (79)	0.375
Male	14 (27)	109 (21)	
Age (year)	80.1 ± 7.9	74.6 ± 10.9	<0.001
Height (m)	1.60 ± 0.09	1.64 ± 0.08	<0.001
Actual body weight (kg)	58.8 ± 12.0	75.6 ± 15.1	<0.001
BMI (kg/m^2^)	22.8 ± 3.9	27.8 ± 5.1	<0.001
Handgrip strength (kg)	12.8 ± 5.3	23.6 ± 10.7	<0.001
Frail scale, median (IQR)	2 (2–4)	2 (1–3)	0.016
SARC-F scores, median (IQR)	5 (3–6)	4 (2–6)	0.047
SPPB, median (IQR)	5 (3–8)	9 (5–11)	0.001
Body composition			
MM_arms_ (kg)	3.5 ± 0.7	4.6 ± 1.2	<0.001
MM_legs_ (kg)	10.9 ± 2.0	14.2 ± 3.0	<0.001
AMM (kg)	14.4 ± 2.6	18.8 ± 4.0	<0.001
RSMI (kg/m^2^)	5.6 ± 0.8	6.9 ± 1.1	<0.001
Body fat (%)	35.6 ± 7.7	40.3 ± 8.0	<0.001
Bone mass density			
T-score	−2.7 ± 1.1	−1.8 ± 1.3	<0.001

SPPB, short physical performance battery; MM_arms_, muscle mass of the arms; MM_legs_, muscle mass of the legs; AMM, appendicular muscle mass (muscle mass of the arms+ muscle mass of the legs); RSMI, relative skeletal muscle mass index. Values are given as mean ± SD, median (interquartile range), or number (%).

**Table 3:** Comparison of patients with low and normal BMD.

	Low BMD n = 435 (76%)	Normal BMD n = 137 (24%)	*p* Value
Gender (n, %)			
Female	346 (80)	103 (75)	0.285
Male	89 (20)	34 (25)	
Age (year)	76.4 ± 10.3	71.1 ± 11.4	<0.001
Height (m)	1.63 ± 0.08	1.67 ± 0.09	<0.001
Actual body weight (kg)	71.5 ± 15.3	81.9 ± 13.7	<0.001
BMI (kg/m^2^)	26.7 ± 5.3	29.4 ± 4.6	<0.001
Handgrip strength (kg)	21.7 ± 10.3	25.6 ± 11.7	0.001
Frail scale, Median (IQR)	2 (1–3)	2 (1–3)	0.263
SARC-F scores, Median (IQR)	4 (2–6)	4 (2–5)	0.164
SPPB, Median (IQR)	9 (5–10)	9 (6–10)	0.509
Body composition			
MM_arms_ (kg)	4.3 ± 1.2	5.0 ± 1.3	<0.001
MM_legs_ (kg)	13.5 ± 2.8	15.3 ± 3.2	<0.001
AMM (kg)	17.8 ± 3.8	20.4 ± 4.3	<0.001
RSMI (kg/m^2^)	6.6 ± 1.1	7.3 ± 1.2	<0.001
Body fat (%)	39.2 ± 8.2	41.8 ± 7.2	<0.001
Bone mass density			
T-score	−2.4 ± 0.8	−0.1 ± 0.9	<0.001
Previous known osteoporosis			
Yes	99 (23)	12 (9)	<0.001
No	328 (77)	125 (91)	

BMD, bone mineral density; SPPB, short physical performance battery; MM_arms_, muscle mass of the arms; MM_legs_, muscle mass of the legs; AMM, appendicular muscle mass (muscle mass of the arms + muscle mass of the legs); RSMI, relative skeletal muscle mass index. Values are given as mean ± SD, median (interquartile range), or number (%).

**Table 4:** Comparison of sarcopenic and nonsarcopenic patients with normal and low BMD.

	Sarcopenia (n = 52)		*p* Value	Nonsarcopenia (n = 520)		*p* Value
	Low BMD n = 47 (90%)	Normal BMD n = 5 (10%)		Low BMD n = 388 (75%)	Normal BMD n = 132 (25%)	
Gender (n, %)						
Female	36 (77)	2 (40)	0.114	310 (80)	101 (77)	0.458
Male	11 (23)	3 (60)		78 (20)	31 (23)	
Age (year)	80.8 ± 7.7	73.8 ± 7.7	0.113	75.9 ± 10.4	71.0 ± 11.6	<0.001
Height (m)	1.59 ± 0.08	1.67 ± 0.15	0.330	1.64 ± 0.08	1.67 ± 0.09	0.002
Actual body weight (kg)	56.9 ± 9.8	78.8 ± 13.0	0.017	73.3 ± 14.9	82.0 ± 13.8	<0.001
BMI (kg/m^2^)	22.1 ± 3.2	28.3 ± 5.5	0.065	27.2 ± 5.2	29.4 ± 4.6	<0.001
Handgrip strength (kg)	12.9 ± 5.5	12.3 ± 3.2	0.726	22.8 ± 10.2	26.1 ± 11.6	0.004
Frail scale, median (IQR)	3 (2–4)	2 (1–4)	0.975	2 (1–3)	2 (1–3)	0.384
SARC-F scores, median (IQR)	5 (3–6)	5 (1–6)	0.547	4 (2–6)	4 (2–5)	0.281
SPPB, median (IQR)	5 (4–8)	5 (3–11)	0.867	9 (5–11)	9 (6–11)	0.847
Body composition						
MM_arms_ (kg)	3.4 ± 0.7	4.3 ± 0.7	0.046	4.4 ± 1.2	5.1 ± 1.4	<0.001
MM_legs_ (kg)	10.8 ± 1.9	12.6 ± 1.8	0.083	13.8 ± 2.9	15.4 ± 3.2	<0.001
AMM (kg)	14.2 ± 2.5	16.9 ± 2.4	0.065	18.2 ± 3.7	20.5 ± 4.3	<0.001
RSMI (kg/m^2^)	5.5 ± 0.8	6.2 ± 0.3	0.009	6.8 ± 1.1	7.3 ± 1.1	<0.001
Body fat (%)	35.0 ± 7.4	41.1 ± 9.6	0.239	39.7 ± 8.2	41.9 ± 7.1	0.004
Bone mass density						
T-score	−2.9± 0.8	−0.6 ± 0.6	<0.001	−2.3± 0.8	−0.1 ± 0.9	<0.001

BMD, bone mineral density; SPPB, short physical performance battery; MM_arms_, muscle mass of the arms; MM_legs_, muscle mass of the legs; AMM, appendicular muscle mass (muscle mass of the arms + muscle mass of the legs); RSMI, relative skeletal muscle mass index. Values are given as mean ± SD, median (interquartile range), or number (%).

**Table 5:** Comparison of patients with and without osteosarcopenia.

	Osteosarcopenia n = 47 (8%)	No Osteosarcopenia n= 525 (92%)	*p* Value
Gender (n, %)			
Female	36 (77)	413 (79)	0.713
Male	11 (23)	112 (21)	
Age (year)	80.8 ± 7.7	74.6 ± 10.9	<0.001
Height (m)	1.59 ± 0.08	1.64 ± 0.08	<0.001
Actual body weight (kg)	56.6 ± 9.8	75.6 ± 15.1	<0.001
BMI (kg/m^2^)	22.1 ± 3.2	27.8 ± 5.1	<0.001
Handgrip strength (kg)	12.9 ± 5.5	23.5 ± 10.7	<0.001
Frail scale, median (IQR)	3 (2–4)	2 (1–3)	0.020
SARC-F scores, median (IQR)	5 (3–6)	4 (2–6)	0.037
SPPB, median (IQR)	5 (4–8)	9 (5–11)	0.001
Body composition			
MM_arms_ (kg)	3.4 ± 0.7	4.6 ± 1.2	<0.001
MM_legs_ (kg)	10.8 ± 1.9	14.2 ± 3.0	<0.001
AMM (kg)	14.2 ± 2.5	18.8 ± 4.0	<0.001
RSMI (kg/m^2^)	5.5 ± 0.8	6.9 ± 1.1	<0.001
Body fat (%)	35.0 ± 7.4	40.3 ± 8.0	<0.001
Bone mass density			
T-score	−2.9 ± 0.8	−1.7 ± 1.3	<0.001
Previous known osteoporosis			
Yes	14 (31)	97 (19)	0.051
No	31 (69)	422 (81)	

BMD, bone mineral density; SPPB, short physical performance battery; MM_arms_, muscle mass of the arms; MM_legs_, muscle mass of the legs; AMM, appendicular muscle mass (muscle mass of the arms + muscle mass of the legs); RSMI, relative skeletal muscle mass index. Values are given as mean ± SD, median (interquartile range), or number (%).

## Data Availability

The datasets analyzed during the current study are available from the corresponding author on reasonable request.
